# Like Water, We Re-Member: A Conceptual Model of Identity (Re)formation through Cultural Reclamation for Indigenous Peoples of Mexico in the United States

**DOI:** 10.3390/genealogy7040090

**Published:** 2023-11-20

**Authors:** My Ngoc To, Ramona Beltrán, Annie Zean Dunbar, Miriam G. Valdovinos, Blanca-Azucena Pacheco, David W. Barillas Chón, Olivia Hunte, Kristina Hulama

**Affiliations:** 1 Graduate School of Social Work, University of Denver, Denver, CO 80033, USA; 2 Chicana/o LatinX Studies Department, University of Northern Colorado, Greeley, CO 80639, USA

**Keywords:** identity, Indigenous Mexican peoples, enculturation, historical trauma, survivance, memory work, Indigineity

## Abstract

**Background:**

Diasporic Indigenous peoples of Mexico living in the United States continue to survive and reclaim their cultures despite multiple disruptions to identity formation resulting from systematic violence and cultural silencing enacted through white settler colonialism in the United States and Mexico. Honoring Indigenous survivance, the authors present a conceptual model of Indigenous identity healing and reformation that mirrors the dynamic qualities of water for Indigenous Mexican peoples living in the United States.

**Methods:**

The conceptual model arose from a ceremony-based, participatory, digital archiving project documenting Indigenous oral histories. The model is illustrated through case analysis of three Indigenous Mexican individuals living in the United States whose stories holistically represent the model’s components.

**Results:**

The case narratives illustrate how Indigenous Mexican identities are (re)formed by moving through the model components of Rift (disconnection from land, culture, and community), Longing (yearning to find what was lost), Reconnecting (reclaiming cultural practices), and Affirmation (strengthening of identity through community), via Reflection (memory work which propels movement through each stage).

**Conclusions:**

Findings suggest that identities can be (re)formed through reclaiming cultural practices and reconnecting with the community. This conceptual model may be useful for further understanding Indigenous Latinx identity development and healing.

## 1. Introduction

### 1.1. Invocation

We begin with an invocation of water. Water heals, nourishes, and replenishes. It shapes and molds and is present in different forms. Even when we cannot see it, it is there. It can be solid as ice or snow. It exists in the air as humidity and within the earth as groundwater and waterways. It can be waves—a living thing. Water can be raging and relentless, telling one to either move or be moved. It can also be gentle—a stream or babbling brook or a meditation. It can carve canyons into the face of mountains. It seeps through things. It helps moss grow. Water preserves across time. The healing within its destruction comes in its power to cleanse. As water fills containers, it re-members itself to take the shape of its vessel yet retains its own essence. Like water, we re-member.

### 1.2. Re-Membering Indigenous Identities

I write to remember.

I make rite (ceremony) to remember.

It is my right to remember.

—Cherríe Moraga (2011)

This paper presents a conceptual model of identity healing pertaining to Indigenous peoples of Mexico who now live in the United States. Using the ocean as a creative metaphor, the model illustrates how these people reclaim and reform their identities through a fluid and dynamic process of re-membering^1^. Settler colonialism, the sociopolitical and economic systems that sustain white supremacy and settler occupation on Indigenous land, has disrupted and crafted damaging definitions of what it means to be Indigenous (Arvin et al. 2013; Clarke and Yellow Bird 2021; Tuck and Yang 2012; Veracini 2011; Wolfe 2006). This historically anchored and ongoing systematic process, whether direct or indirect, has aimed to eliminate Indigenous peoples to gain land and resources, control definitions of Indigenous identities, and suppress social and cultural practices from which they derive meaning (Clarke and Yellow Bird 2021; Simpson 2014; Tuck and Yang 2012; Wolfe 2006). Indigenous Xicanx scholar Susy Zepeda (2022) reminds us of the purposeful nature of erasure that leads to forgetting:

It is a colonial legacy to forget, and it is a response to trauma to have gaps in memory; conversely, it is a practice of decolonization and healing to remember. This is not an easy or linear path to walk.

(p. 17)

Despite how deeply entrenched and incessant settler colonialism runs, it has not been completely successful. Like underground water that remains, resists and will continue to resurface, Indigenous peoples everywhere have survived and continue to resist the daily barrage of systematic efforts to extract land, resources, and culture from their communities (Vizenor 2008). Minnesota Chippewa scholar Gerald Vizenor (2008) calls this “Native survivance,” which he describes as “an active presence over absence, deracination, and oblivion; survivance is the continuance of stories, not a mere reaction, however pertinent” (p. 1). He describes survivance stories as a “renunciation of dominance, detractions, obtrusions, the unbearable sentiments of tragedy, and the legacy of victimry” (Vizenor 2008, p. 1).

As water shapeshifts to fill new containers while remaining true to its elemental form, so do Indigenous peoples who understand that “water is not only life to people, animals, and plants but is also intimately tied to many other phenomena in our world and has deep spiritual meaning” (Clarke and Yellow Bird 2021, p. 88). As one of our co-researchers^2^ says:

The way that we are born… is crucial, and we need to also remember that because we’re carried with water, we’re in water, and that water is a transmitter of emotions… Everything that we say, everything that is, is able to [resonate] in our womb… Generational trauma has been passed on… And the womb, carries the memories of that.

(Yoloteotl)

Yoloteotl’s cultural understanding is supported by mounting evidence that trauma exposure can be transmitted through generations via epigenetic changes in gene expression, which alter DNA methylation (Lehrner and Yehuda 2018; Youssef et al. 2018), and that prenatal and possibly pre-conception conditions of maternal and paternal stress may have particularly salient impacts on their children (Lehrner and Yehuda 2018; Mbiydzenyuy et al. 2022). In addition to these biological mechanisms of intergenerational inheritance, Indigenous people carry cultural mechanisms, such as storytelling, narratives, and traditional practices that also transmit identity, joy, and community, which can be re-membered and reclaimed (Fraser and Michell 2015). Scholars describe memory work as an integral step, not just for challenging systems of oppression but also for supporting healing and liberation from colonial ideologies that disrupt healthy identity development (Clarke and Yellow Bird 2021; Moraga 2011; Zepeda 2022). Our model builds on existing cultural identity literature by operationalizing specific yet fluid ways people within diasporic Indigenous communities may repair, reclaim, restore, and create new identities.

Using three oral histories from a ceremony-based participatory research (Cer-BPR) (Valdovinos et al. 2022) project, the authors use a case study analytical approach to document and illustrate a conceptual model that captures how healing can occur in Indigenous Latinx communities^3^ who have experienced multiple disruptions to their place, land, culture, and language as it impacts their sense of identity. We aim not to identify parameters of Indigenous identity but to explore how these communities conceptualize and experience what it means to be Indigenous.

In alignment with Indigenous research principles of respect, reciprocity, and relational accountability (Wilson 2008), we begin by sharing our unique and diverse positionalities as authors to contextualize who we are in relation to the project’s co-researchers, communities, and questions. Accordingly, we situate our individual and collective ancestral histories of displacement, diaspora, reconnection, and restoration in conversation with our participants, their stories, and the model of Indigenous identity healing and restoration that has emerged.

United by a digital archiving project that preserves Indigenous oral histories, we come together with identities that converge, intersect, and depart from one another. We are Black, Afro-Caribbean, of Indigenous Mexican descent, Xicana, Vietnamese American, of P’urepecha descent, Xinka, mixed race, Maya Poqomam, Native Hawaiian, Samoan, immigrant, first-generation, generation 1.5, third generation, cis-gendered, queer, able-bodied, women, men, and femmes. Our ancestors and our genetic memory have ties to (what are now called) Northern Mexico, Southwest United States, Guatemala, St. Lucia, Vietnam, Liberia, Hawai’i, Samoa, and Northern Ireland. We are also mothers, fathers, parents, daughters, sons, aunties, uncles, sisters, and relatives. Our ancestors have sought sustenance from, navigated, and traversed (forcibly and by choice) the Pacific and Atlantic Oceans and the Caribbean and Mediterranean Seas. Like waves making their way to shorelines, we have arrived here as social workers, educators, and students, reconciling and healing our own identities in the context of historical and contemporary settler colonial projects.

## 2. Literature Review

### 2.1. Settler Colonialism and Indigenous Identity

White colonial heteropatriarchy has disrupted Indigenous peoples’ ability to know ourselves, our places, each other, and the cosmos (Arvin et al. 2013). Settler colonialism, as explained by Wolfe (2006), is the economic and political foundation designed to replace and reconfigure Indigenous land into settler property (Rowe and Tuck 2017; Tuck and Yang 2012; Wolfe 2006). White settler colonialism has inflicted multi-level mass violence on Indigenous peoples through land theft, genocide, eradication of cultural traditions, and systematic assimilation strategies and policies (Clarke and Yellow Bird 2021; Evans-Campbell 2008; Walters et al. 2011; Walters and Simoni 2002). Beyond the violence imposed on Indigenous peoples’ bodies, settler colonialism has created systems of individual and collective amnesia that inhibit the spiritual customs, cultural practices, and histories of Indigenous peoples while refusing to acknowledge any past violence (Clarke and Yellow Bird 2021; Kovats Sánchez 2020; Zepeda 2022). This amnesia results in an erosion of the self where Indigenous peoples may be hesitant or unable to remember how to be Indigenous due to these painful historical and continual processes (Brant 1994; Zepeda 2022). The shame or internalized oppression toward one’s indigeneity has been noted as a psychological response to historical traumas (Evans-Campbell 2008; Orozco-Figueroa 2021; Sotero 2006; Weaver 2001).

Systems of racial orders and racialized authenticity criteria were imported from European colonial racialization projects (Robinson 2020) and reinscribed in the United States through federal Indian policy (Weaver 2001). For Indigenous peoples in the United States, the notion of a “real Indian” often leads to internalization and external policing of imposed criteria related to blood quantum, federal recognition, tribal enrollment status, phenotype, and performance of culture in Indigenous communities (McKay 2021; Saldaña-Portillo 2017; Weaver 2001). Institutionalized Indigenous legitimacy criteria have led to persistent contestation between and within Indigenous communities who enact criteria for citizenship and belonging to exercise their sovereignty (Cotera and Saldaña-Portillo 2014; Leza 2019; McKay 2021). The combination of settler colonial erasure, cultural amnesia, imposed authenticity criteria, and the importance of tribal self-determination and sovereignty have made defining Indigenous identity in the United States complex and precarious.

Defining indigeneity becomes even more complex when considering diasporic Indigenous communities because different countries impose similar colonial violence but varied inclusion and exclusion criteria for authenticity and belonging (Navarrete 2016). For instance, the Constitution of Mexico, where the three case studies originate, defines Indigenous communities as “the community that constitutes a cultural, economic and social unit settled in a territory and that recognizes its own authorities, according to their customs” (United Mexican States 2015). No national definitions of Indigenous identity exist outside of this constitutional description other than protected status descriptions within each Mexican state (Flannigan 2016). Furthermore, prior to 2000, the Mexican government census utilized linguistic criteria for identifying Indigenous peoples. Since 2000, the census has added self-identification, or a self-reported sense of belonging to an Indigenous community, as another criterion independent of language (Gonzalez 2018). As such, the common practice of self-identification rests upon a politically volatile backdrop where legitimacy is also contested in the public sphere (López Caballero 2021).

The complexity of Indigenous identity in Mexico stems in part from the country’s history of colonization starting some 500 years ago by the Spanish, who sought to erase Indigenous identities through methods such as implementing a casta system in some parts of the country, which stratified status by skin color (Moreno Figueroa 2010). Later, the Mexican government employed the concept of mestizaje, which removed tribal identities to create a Mexican national identity as a mix of Spanish and Indian. The legacy of Spanish racialized systems in Mexico endures through persistent anti-Indigenous and anti-Black racism (Moreno Figueroa 2010; Navarrete 2016). In their work on critical Latinx Indigeneities, Blackwell et al. (2017) propose that racial hierarchies from Latin America have fused with the racial hierarchies of the United States to impact lived experiences and identity perceptions of Indigenous immigrant families and communities. This hybridity may compound experiences of discrimination in these communities, both in Mexico and the United States. In Mexico, some Indigenous people opt out of disclosing their Indigenous origins due to disconnection from their original lands and communities, while in the United States, Indigenous peoples of Mexico may engage in a strategic process of being *oculta/o/e* (hidden) or invisible (Machado-Casas 2012) due to ongoing anti-Indigenous racism from their Mexican-descent compatriots and other Latinxs (Barillas Chón 2021). In the United States, Indigenous communities from Mexico are often relegated to the margins within multiple national, racial, and ethnic spaces (Casanova et al. 2016; Kovats Sánchez 2020); ethnographic studies have documented anti-Indigenous discrimination from mestizo peers within Indigenous Mexican youth living in the United States (Barillas Chón 2010).

These intersecting and multifaceted identities are further complicated by diverse migration histories shaped by the policies that lead to displacement, such as the North American Free Trade Agreement (NAFTA) and sustained structural inequity and lack of access to resources (Casanova et al. 2016; Stavenhagen 2015). Complexities in identity experiences are particularly salient as the population of Indigenous communities in the United States with roots in Mexico as well as Central and South American continues to grow (U.S. Census Bureau 2020). For example, In 2020, “Mexican American Indian” (MAI) became the largest tribal grouping census category in the United States (Contreras and Reyes 2021; U.S. Census Bureau 2020). When the experiences of identity are as multidimensional as the lands from which these communities originate, and when these same communities resist the acceptance of racial, ethnic, or tribal categories created and imposed by historical and current settlers and colonial governments, it can be difficult for these communities to summarize their identities. Thus, the available literature describing the identity experiences of Indigenous peoples of Mexico living in the United States is emergent (Barillas Chón 2010; Casanova et al. 2016; Gonzalez 2018; Kovats Sánchez 2020; Nicolas 2021); we have found no models of identity development that are specific to the unique diasporic and urban United States based experience described by our participants.

### 2.2. Towards a New Model of Identity (Re)formation

Indigenous communities living with contested identity labels and a diverse range of related experiences share commonalities in how they maintain and transport connections to land, culture, and memory and create their own ways of preserving and passing on cultural ties and practices (Alberto 2017; Fernandez and Beltrán 2022; Casanova et al. 2016). For Indigenous peoples, connection to their original land and place is central to identity, self, purpose, and overall worldview (Walters et al. 2011; Beltrán et al. 2018; Casanova et al. 2016; Clarke and Yellow Bird 2021; Fernandez 2019). Cultivating spaces of connection and practicing place-based traditions in new spaces has helped communities maintain a connection to their original lands while resisting forces of assimilation and colonization (Fernandez and Beltrán 2022; Casanova et al. 2016; Fernandez 2019). For Indigenous people disconnected from family, culture, tribe, or community, enculturation, reclamation of Indigenous languages and traditions, and positive cultural identity promote health and well-being and identity formation (Beltrán et al. 2020; Fernandez and Beltrán 2022; Beltrán et al. 2023; Evans-Campbell 2008; Fernandez 2019; Gone 2011; Masotti et al. 2023; Walters and Simoni 2002). In many cases, Indigenous peoples are not only reclaiming and preserving their identities but are also creating new definitions, traditions, and practices in an effort to simultaneously re-member what was lost (Fernandez and Beltrán 2022; Beltrán et al. 2023; Kovats Sánchez 2020; Zepeda 2022) and envision their own futures (Beltrán 2023; Dillon 2016; Fricke 2019).

## 3. Methods

The conceptual model we present emerges from oral history interview data collected through a ceremony-based participatory digital archive project that centers traditional Indigenous health knowledge of Native American and Indigenous Latinx people in urban areas of a western region of the United States. The project was funded by the National Institutes of Health and the National Libraries of Medicine (5G08LM013186) and received IRB approval (1122295). After multi-level informed consent was obtained, co-researchers engaged in in-depth oral history interviews designed to reflect components of an adapted southwestern (Mexica) medicine wheel. Guided by storytelling principles (Archibald 2008) and witnessing methodology (Beltrán 2023; Iseke 2014; Valdovinos et al. 2022), the oral histories consisted of open-ended questions on origin, health and medicine, birth and death, roles, dreams, and self-perception. All interviews were video- and audio-recorded and were anywhere from 2 to 6 h in duration. Nineteen initial interviews were conducted in person, followed by 11 interviews conducted online due to the COVID-19 pandemic.

### 3.1. Demographics

Our research team is led by two Indigenous Latinx professors. All members of our research staff have been Indigenous, Native American, Latinx, Black, or people of color with expertise in Indigenous and culturally grounded qualitative research methodologies. All aspects of the project were overseen by a Community Advisory Board (CAB) composed of Native American and Indigenous Latinx community leaders with cultural and professional knowledge in mental health, k-12 education, curriculum development in higher education, HIV and health disparity advocacy, Tribal law, Tribal education, environmental justice, social work, and culturally responsive program management.

The study’s pilot phase used a convenience sample strategy to conduct 10 initial oral histories with graduate students, staff, and faculty within our university and associated cultural affinity community who identified as Indigenous/Latinx. The pilot sample was further used to evaluate and refine our study protocol and questionnaire. The second phase of data collection utilized a mixed strategy of convenience and snowball sampling via social media and email listservs to recruit an additional 20 co-researchers from university organizations, cultural affinity community groups, participant social networks, and professional and community networks of CAB members. All co-researchers were adults (18+) who identified as Native American or Indigenous Latinx and lived in urban areas of a western region of the United States. Of the total 30 co-researchers, 26 completed the demographic questionnaire. Their ages ranged from 19 to 65 (mean = 41.7), and 46% (*n* = 12) identified as cis-gender women, 23% (*n* = 6) as cis-gender men, and 16% (*n* = 4) as gender non-conforming, non-binary, or two-spirit. Two reported that the categories did not reflect their gender identity, one participant identified as trans-male, and one participant selected preferred not to say.

Demographic questions related to race and ethnicity were designed to account for intersectional race/ethnicity and Indigenous or tribal identities. Eighty-eight percent (*n* = 23) identified as Native American or Indigenous, and within this group, 15 identified as both Latinx and Indigenous. Three additional people identified as Latinx alone. When asked about specific identity terms, participants identified as Chicano/a, Indigenous/Indigena, Mexican, Native, and/or Xicana. Participants further described their tribal heritage as Apache, Andina, Aztec, Chicana, Comanche, Diné/Navajo, Genízaro, Indeh, Lakota, Mexica, Ñ’uu Savi, Otomi, Poqomam, P’urépecha, Taino, Tihua, Tehua, Ute, and Yaqui.

### 3.2. Conceptual Model Development and Data Analysis

#### 3.2.1. Data Immersion

The analysis began with data immersion, which is often considered an important first step of qualitative analysis as it allows the researcher to become familiar with a holistic impression of the full dataset (Creswell 2013; Forman and Damschroder 2007; Green et al. 2007; Linneberg and Korsgaard 2019). Aligned with Indigenous research methods, data immersion creates an opportunity for research team members to deepen their relationship with the stories and testimonies shared by participants (Hunter et al. 2002; Wilson 2008). Without the structure of a codebook or analytical software, immersion encourages attentiveness to participants’ affect, tone, time to response, and interviewer/participant relational dynamics (Green et al. 2007; Linneberg and Korsgaard 2019).

Following immersion, we conducted first-level, structured coding, which reduced the data into salient themes (Saldana 2021); we then used Mediaspace video-editing software to cut the interviews into smaller clips representing thematic categories identified during initial coding. Clips were organized in an Excel spreadsheet based on themes, descriptions, memos, and timestamps of individual stories. Upon reviewing this initial dataset, Author 1 identified the five reflexive experience categories that occurred across the data. Through continued analysis involving the full research team, we identified these reflexive themes throughout the sample and designed a representative conceptual model from these data.

Struck by how the experiences seemed reflexive and unbounded, we immediately drew a connection between the model and the physical and other life-giving properties of water as they are understood in Indigenous epistemologies (Clarke and Yellow Bird 2021; George and Wiebe 2020; Yazzie and Baldy 2018). Later, while watching an educational program with her young children that described the layers and qualities of the ocean depths, Author 2 recognized a further connection between the way the co-researchers’ identity experiences may metaphorically relate to the changes in light, pressure, and life sustainability across the ocean layers. It is important to note that we metaphorize the five ocean layers (O’Leary and Roberts 2018; Woods Hole Oceanographic Institution n.d.) with a focus on the simple observation that the ocean has gradients of light, pressure, and organic life across its depths. We use this metaphor, additionally, to reflect the Indigenous epistemology related to the deep interconnected relationships between people and every aspect of the earth’s ecosystems. The popular discourse “water is life” illustrates Native and Indigenous epistemologies in the fight for water rights. Yazzie and Baldy (2018) describe these deep interconnections:

Water runs through our human veins and connects us to everything. The water we drink is the water the salmon breathes, is the water the trees need, is the water where the Bear bathes, is the water where the rocks settle. Many of our stories foreground relationships to water. These stories show us that water is theory; theory that is built from relationships to the land, the earth, everything.

(p. 1)

Data immersion continued for six months with weekly meetings with the project research team and involved listening to 70 h of audio-visual interview footage in designated teams of 2 to 3 researchers, which facilitated our process of updating the model and reflecting on our shared findings. The researchers simultaneously participated in self-reflexivity by keeping informal memos and journals to document any affective, cultural, or relational experiences that arose while listening deeply (Birks et al. 2008).

#### 3.2.2. Case Analysis

Qualitative case study methodology is an appropriate strategy for exploring phenomena such as lived experiences, events, and the contexts in which they occur (Houghton et al. 2014; Miles and Huberman 1994). Within-case analysis is useful for providing a deep dive into specific themes and details within each case (Houghton et al. 2014). Seawright and Gerring (2008) define case studies as an “intensive (qualitative or quantitative) study of a single unit or small number of units (the cases), where the researcher’s goal is to understand a larger class of similar units” (p. 296). Following their protocol for selecting case studies within a larger sample, we chose these three narratives using both Typical selection, wherein their similarities across cases affirm the model components, and Diversity selection, wherein differences across narratives display the range of experience across model components. Thus, although the conceptual model was present in various ways across all interviews, we chose to conduct an exploratory, descriptive, case study analysis of these three oral histories to concretely illustrate the five reflexive identity process categories with depth and breadth. Our aims in this paper are to propose this model with exemplar data that fully illustrate the components of the model; systematic analysis and presentation of this model across our larger dataset will be detailed in future works.

With the initial conceptual model developed, we began our case analysis by first using video editing software to clip interviews from each oral history into sections that reflect themes related to the model. A detailed Excel spread sheet was used to organize selected clips corresponding to emergent codes and subcodes. Clips were then transcribed verbatim and uploaded to Dedoose analytical software for further coding and organizing. After all transcripts were coded, we consolidated the codes applied by each member through interrater reliability checks that determined which aspects of the model were best reflected in each transcript.

Aligned with our values of relational accountability (Wilson 2008) in the research process, we presented the final model to CAB members who affirmed the application to their own individual, professional, and community experiences. After the presentation, one CAB member requested that we present the model to an Indigenous youth in a non-profit organization setting because of its applicability. For member checking, we also shared the draft manuscript with the co-researchers to confirm that the presentation of their stories matched their understanding. We highlighted their exact quotes and the narrative that described their personal stories and asked that they review them for accuracy, appropriate analysis, and description of their stories. We received affirmation of our analysis and presentation. After adjusting certain details of their stories to protect their privacy, we also asked them to confirm which aspects of their stories they wanted changed or maintained.

The three co-researcher narratives come from Yoloteotl, who identifies as Aztec and Chichimeca, was born in Mexico and grew up living between the United States, Canada, and Mexico; Olga, who is Yaqui and Otomí from Guanajuato, Mexico and moved to the United States at age four; and Bonifacio, who is Ñ’uu Savi from Oaxaca, Mexico and moved to the United States at age seven. All co-researchers requested that we use their real names. As depicted in the model below, within each of their stories are multiple examples illustrating the dynamic process of reforming their Indigenous identities through remembering and reclaiming.

## 4. A Conceptual Model of Identity Reclamation

**Figure 1.**
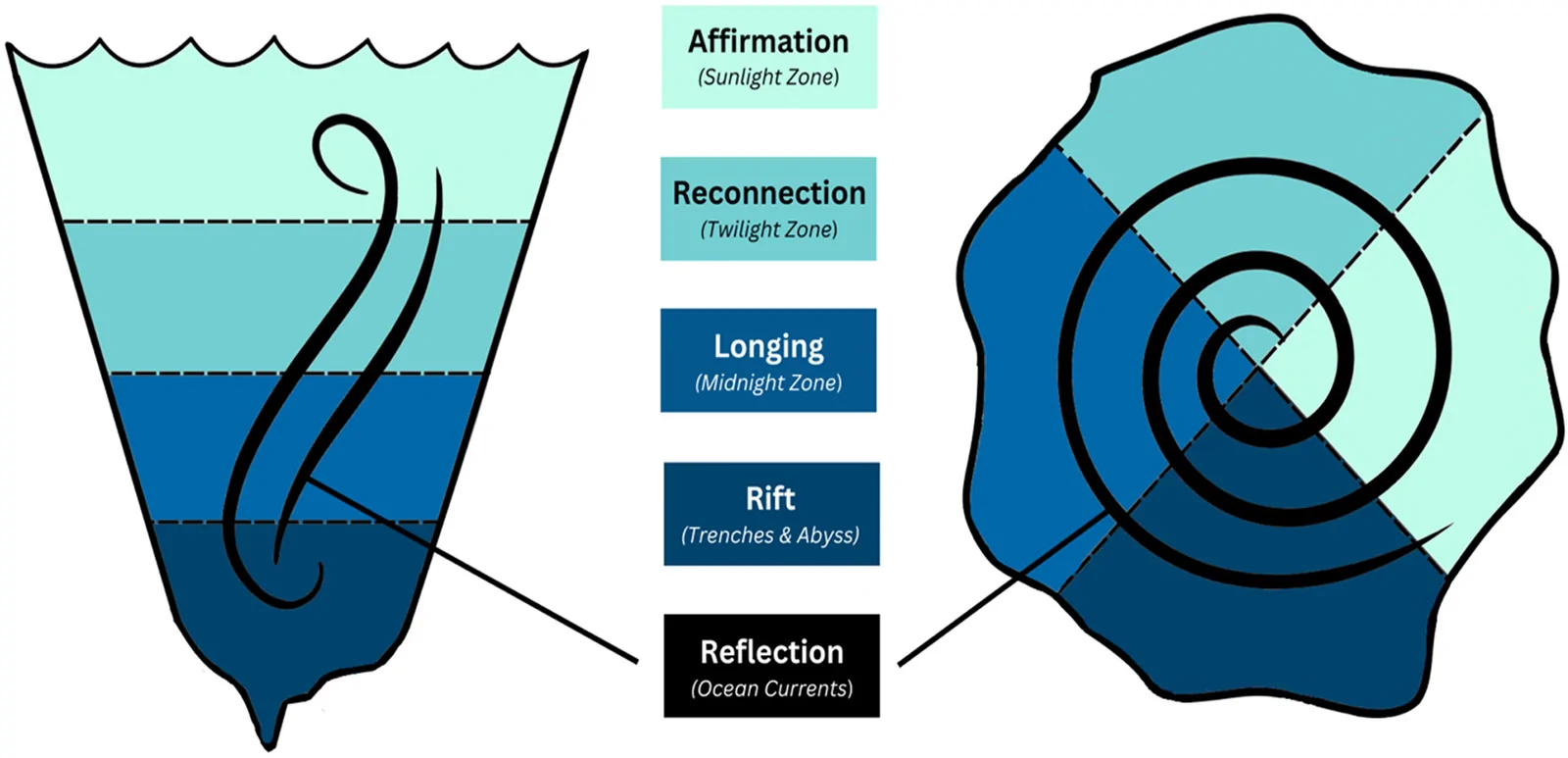
Model of Identity (Re)formation through Cultural Reclamation.

The model we present reflects the properties of the ocean as a metaphor, with its ebbs and flows, depth and breadth, and variations of light and pressure. The model consists of five components: Reflection, Rift, Longing, Reconnection, and Affirmation, which are mapped onto the layers of the ocean^4^ (see Figure 1).

In this figure, identity is represented by the ocean, as viewed from the side (left) and also from above (right). The different layers or sections of the ocean reflect different components of the model: *Rift*, *Longing*, *Reconnection*, and *Affirmation*. *Reflection* is represented by swirls and spirals which move across each part of the model.

The layers of the ocean have no hard boundaries or borders but rather variations of light filtration, temperature, and capacity to support different forms of life (O’Leary and Roberts 2018). These layers act as metaphors for how experiences and knowledge of culture, land, community, and self can be affected by light (seeing/remembering/learning), temperature (warmth/connection to community), and pressure (feeling a sense of isolation or liberation through seeing oneself in others and being seen). At first glance, the ocean metaphor may not appear to align with Indigenous ontologies and epistemologies emerging from connections to land (McCoy et al. 2017), the land-locked area where this study took place, or these participants’ lands of origin and ancestry. However, as we consider how land emerges from the ocean, how the ocean serves as the life source for the earth, and how the majority of the earth’s body, similar to our own bodies, is composed of water, we arrive at a deeper interconnection between the ocean and Indigenous ways of knowing and being (George and Wiebe 2020; Grande 2015; Yazzie and Baldy 2018).

This model also illustrates an organic cycle of identity reformation, where different experiences may flow into one another. Co-researchers may repeat this cycle multiple times in their lives, experience some components in varying orders, or even experience multiple elements simultaneously. Although our model is multidimensional and cyclical, for ease of presentation, we present each of its components through a narrative arc that moves from colonization/disconnection to decolonization/connection and liberation. As part of the cycle of creation, there is no end point throughout this model and no such thing as a sickness that can be “healed,” just the ongoing pathway of identity healing that is alive, dynamic, and present.

### 4.1. Reflection—Ocean Currents

Ocean currents flow through every layer of the ocean in the form of internal waves, large-scale vertical movement of ocean water, and circulation of global waves driven by fluctuations in temperature and salinity (Garrison 2012). Similarly, *Reflection* is dynamic and moves across each part of this model. The narratives presented here arose from the interview guide for oral history, which invited participants to reflect continuously on different aspects of their lives and familial experiences. In the model, *Reflection* works like the currents within the ocean; it leads to shifts. It is movement. This stirring of the mind, body, and spirit begins the memory work and propels one through each stage of the model toward identity reclamation.

### 4.2. Rift—Trenches and Abyss

Trenches, the deepest part of the ocean, are formed by one tectonic plate sliding beneath another to create steep canyons (Garrison 2012). Although nothing is illuminated here in the deepest, coldest regions, there are glimmers. There is life—small organisms living at this depth have adapted to life in the immense pressure and complete darkness. In our model, this is the site where lost memories and historical experiences lie. Here, we use *Rift* as a conceptual tool to visualize and think through the violent experiences of disconnection and displacement between self and Indigenous culture resulting from settler colonialism. It is not used in a damage-centered manner (Tuck 2009) but rather to analyze the various practices by individuals, families, and communities to mend disconnections, cracks, and ruptures that affect how they perceive and define their identities. *Rift* occurs in various ways and across multiple instances over time. *Historical rifts* are often embedded in sociopolitical history, which serves as context for those rifts that have occurred. Some, but not all, historical *Rifts* begin with ruptures between people from their original land and culture (e.g., colonization, displacement, relocation, or migration). The separation from land can also send reverberations that morph into other types of *Rifts* across generations.

Social *Rifts* occur when relations to the land and community members are disrupted or lost or when there is internal conflict within a community about who within that community gets to claim a certain identity. Separation from land and place can also lead to cultural and linguistic *Rifts* where one must adopt new cultures and languages, often while suppressing or hiding one’s original customs and languages, to adapt and survive. Even if one resides in or is still connected to one’s original homeland, there can be an experience of ancestral *Rift* wherein the flow of intergenerational knowledge and communication about identities is disrupted. Coupled with these outward, physical, and relational *Rifts* are often internal and psychological in nature; they may manifest as pushing away Indigenous knowledge, separation, othering, and shame—survival strategies that may be passed down for generations (Caminero-Santangelo 2004; Sotero 2006).

When *Reflection* moves through a *Rift* zone, one can experience recognition that a *Rift* has occurred. In this dynamic process, one becomes aware of the cause of the *Rift*(s). Within the trenches, *Reflection* brings words and pictures to what previously existed only in shadow and may start to shift out of it. The previous sense of “I simply don’t know,” in the trenches of the ocean has now shifted into a sense of “I know that I don’t know.” Having recognized the Rift, one rises up out of the steep canyons and onto the Abyss level, marked with more level ground and greater movement of organisms and geologic activity (Woods Hole Oceanographic Institution n.d.). It is that glimmer in the deep and dark—the spark–that invites more knowing.

### 4.3. Longing—Midnight Zone

Moving up from the Trenches, we enter the Midnight Zone, where *Longing* takes place. In *Longing*, one carries an intuitive knowing that manifests as a desire to reconnect with what was missing due to *Rifts*, such as medicinal practices, family genealogy, and ancestral wisdom. This phase is marked by an active sense in one’s mind, body, and spirit that something is missing or incomplete about what one understands about one’s own identity and culture. The strong sentiment in this phase is, “I long to belong, I long for connection.” It is an embodied stage, and the *Longing* manifests as sensations and emotions in the body, which can include but are not limited to pain, resentment, grief—including ancestral grief, the psychological suffering from unresolved grief from previous generations (Heart and Chase 2016)—and, importantly, joy, which has endured despite legacies of colonization. Reflecting upon one’s *Longing* can drive one to take action toward finding, once again, what was erased and left unknown. Such steps lead one to the next phase of *Reconnecting*, which ultimately leads one to resurface toward a place of belonging again.

### 4.4. Reconnecting—Twilight Zone

Moving upward, we enter the Twilight Zone, where the first faint rays of sunlight begin to shimmer (Garrison 2012) as one begins the process of *Reconnecting*. In this phase, one makes an active, individual choice to reconnect with what was previously lost. Driven by a sense of agency and self-determination, one actively chooses to reclaim aspects of culture and identity. In other words, one moves with the sense of, “I know what has been missing, and I want to return—I choose to return and remember.” *Reconnecting* can sometimes arise out of ancestral memory, or “genetic memory” passed down through generations (Fernandez 2019; Lim and Brunet 2013), which guides one to search and find even if there is no conscious recollection of what was lost. In some cases, the memory of what was lost runs through generations and resurges in a latter generation as they re-engage with cultural practices. *Reflection* within the *Reconnection* zone allows one to process the impact of *Reconnection* on one’s own identity. This is often, but not always, experienced as powerful and positive, wherein one feels a renewed sense of identity. As identity re-forms through cultural reclamation, one feels inspired to keep swimming up toward more light.

### 4.5. Affirmation—Sunlight Zone

The light emerging from the Twilight Zone grows and amplifies as one moves further up toward the Sunlight Zone, where *Affirmation* takes place. In this zone, a new, positive sense of identity encountered from *Reconnection* is maintained and reinforced through community, activism, reimagining possibility, and envisioning futures. *Affirmation* moves beyond the individual; it exists at a community level and occurs when one receives positive feedback from other community members that confirms the value of cultural *Reconnection* that fosters collective post-traumatic growth (Ortega-Williams et al. 2021). As one engages with reflection within the *Affirmation* zone, there is a strong sense of connection and safety within the community, and one develops the sense of “I belong.” As one gets closer to the surface of the water, the light spreads and brightens until, at some point, one fully resurfaces and can breathe. Next, we share stories from three of our co-researchers whose journeys illustrate the movements of the model. As we describe the arc of their stories, their own words are used throughout.

## 5. Case Narratives

### 5.1. Bonifacio

#### 5.1.1. Rift

Bonifacio was born in the southern region of Oaxaca, Mexico, and is a member of the Ñuu Savi tribe. *Historical Rifts* that affected his family and community began with the Spanish Conquest, where colonizers renamed their language the “language of the poor” to “humiliate” them. In the 1970s and 1980s, the Mexican government required his tribal community to adopt Spanish or Christian names instead of their original Indigenous names “to get [government] benefits.” Bonifacio’s account maps onto the Mexican government’s colonial efforts in the 20th century to erase Indigenous identities—often through using Spanish names and surnames to replace their original Indigenous names (Denham 2023). In the 1990s, a *Rift* from land occurred when Bonifacio’s family was “forced to migrate from my village because NAFTA made [our] crops not worth anything,” and “it was pointless for my parents to stay there and buy corn from this company, cultivate, and then give it back.” While American policymakers viewed NAFTA as a political triumph that would loosen economic trade policies and make agriculture more affordable, NAFTA often impacted small rural farming communities, many of whom were Indigenous, by pricing them out of the market entirely (Stavenhagen 2015).

As his family was forced to relocate due to financial hardship resulting from NAFTA’s impact, the displacement led Bonifacio to new lands in Mexico and later the United States. As a result, he experienced multiple cultural and linguistic *Rifts* throughout his life. When he was five, his family moved to a northern part of Mexico and enrolled him in a Spanish-speaking school where other kids made fun of him for not knowing Spanish as he still primarily spoke his tribal language. During this time, his dad moved to California to work in seasonal agriculture, where a family friend suggested that the rest of his family also come to the United States.

When he was seven, Bonifacio’s family moved to the Pacific Northwest, where there was better-paying seasonal work. Bonifacio reflects how “that was the first time that I’d seen a bunch of white people [and] kids.” Again, he found himself having to learn a new language, English, and questioned why he “had to learn about all these languages.” In middle school, he began to question his identity because he was “the only brown kid in class,” and his friends often made fun of him for being Native to the point where he “didn’t want to… be Indigenous.” During his interview, he reflects upon this time as his family “further dislocating” themselves from their culture throughout the journey just because of the way Indigenous people have… always been treated… Not just here in the United States, but also in Mexico.”

#### 5.1.2. Longing

In high school, Bonifacio began to feel differently about his Indigenous identity and longed to connect with an identity that he could feel genuine about. During this time, Bonifacio also began *Longing* to return to his village: “I wanted to go back… I’m going to the mountains there… That’s how I feel.” Feeling angry from the unfair treatment and messages he was issued from society, he described a time when he found temporary *Affirmation* through the “cholo lifestyle” not because he “wanted to be a gangster” but because he “wanted to belong to some type of community.”^5^ “There was a group of us from Oaxaca,” he says, and “we wanted to protect ourselves… our identities.” That was the point where he learned about the struggles of identity within Chicano culture, which led him to reflect on his identity struggles:

… I can’t be a Mixteco or Ñuu Savi here because I’m not in my village. Or I can’t be Mexicano here because I’m not Mexicano. I can’t be white because I don’t look like white. All these identities mix you, it messes up who you want to be and it’s hard for you to choose what you want to be.

#### 5.1.3. Reconnecting

As he was experiencing this *Longing*, Bonifacio reconnected with his identity when he “first realized that I looked like an Indigenous person” during a visit to the mall as a teenager. His friend was trying on clothes and asked him to help find him another size. As he stepped into the fitting room to bring his friend the requested clothing, Bonifacio noticed his reflection in the three-way mirror adjacent to the fitting room. Seeing his reflection and noticing the profile of his Indigenous features “from the side of my face,” it “hit me that I couldn’t hide that.”

A second instance of *Reconnection* occurred during college when he joined the student organization Movimiento Estudiantil Chicano de Aztlán, or M.E.Ch.A (Chicana/o Student Movement of Aztlan). “In a white campus… you always feel like you’re getting pushed out, and you want to get your identity.” Other M.E.Ch.A members affirmed that he belonged just as much as the white students, helping him reflect, “If my parents are working in the fields for me to be here, then… I needed to be here.”

After finishing college and getting married, Bonifacio traveled with his wife to Oaxaca. Although he “hadn’t been there since I was a kid,” an elder in the village approached him and shared that she was his aunt upon learning who his parents were. The woman continued telling him, “Don’t ever forget that this is your land, and you were born here. And when you come here, you need to be here.” She continued to say that many people in his generation “move away from the village,” but “for whatever reason, they don’t come back.” As a result, “there’s no youth [in the village] to pass on the traditions and the culture.” She reminded him that he “need[s] to keep this alive.” These words from the elder helped Bonifacio reconnect further to his Indigenous identity. It planted a seed of commitment that he would learn and cultivate his tribe’s traditional cultural practices as a father to ensure their cultural legacy continues with his children and beyond.

#### 5.1.4. Affirmation

As Bonifacio has worked to keep his culture alive, he reflects on the experience of eating mole as akin to the experience of reclaiming his Indigenous identity:

Even though [mole] is hot, even though…it burns your mouth, you still want to eat more and more… At the end of the day, if you keep eating it, it feels good. I like that feeling. I think for us [Indigenous people in this country], we get all this racism [and] prejudice, we still want to keep our Indigenous [ways of life] alive. It makes us more be, want to be part of that, right?… We have to keep the fire on or else if it’s, if it’s out, who’s going to be able to see again and turn it back on, right?

Motivated to maintain the connection to his identity, Bonifacio moved to a major city with a “huge Chicano history” to contribute to his community and work “against injustice.” He works in a public school, helping kids and their families with socioemotional development.

Bonifacio also maintains his culture in how he has raised his son, starting with giving him a traditional name in his language. He passes on key teachings he learned growing up to his son, such as thinking with his heart, not just his mind, and “putting your heart and soul and intention into what you’re gonna do.” One of the ways he keeps the teachings alive is by growing corn and beans outside their home in a way his grandfather taught him, such as watering the plants with intention. Bonifacio says that his son is “very good at it” because “every time we wake up, he wants to go water [the corn outside]… and it’s kinda teaching him the basics of life… for you to take care of others, you have to take care of yourself first.”

Last, Bonifacio hopes to teach his son that “he comes from multiple different cultures” and that what he needs “to learn who he is first in order for him to be ready to give to the world.” He shares that he wants to take his son back to his homeland and “learn what he wants from that.” He hopes that his son can “know where he was born” and “be proud of how far you can trace [your] roots; see how lucky you are to keep it alive.” With his hopes and continued action, Bonifacio affirms not just his own indigeneity but also that of future generations.

### 5.2. Olga

#### 5.2.1. Rift

Olga was born in the Nuevo León region of Mexico, with roots in Guanajuato and San Miguel de Allende. After migrating to the United States at the age of four with her sibling, she recalls how, upon “starting school in the U.S., they (school administrators) said I had to change my first name” because “it was so long, you couldn’t fit it on the Scantron.” After removing a part of her name that her mom had given her, she reflects on how she has since “lived a life of struggle and sacrifice for the most part where joy was a luxury.”

Though she is Yaqui and Otomí, Olga was unaware of her Indigenous roots growing up, knowing only that she was Mexicana because “that’s what my family said.” In college, she interviewed her grandmother, her dad’s mother, for an assignment in a Mexican history class. During this interview, her grandmother started speaking in her native language, Otomí, which surprised Olga because she “never knew… that [we were] more than just Mexican.” In that interview, Olga learned that her grandmother experienced a lot of shame, discrimination, and oppression around her Indigenous identity “to the point that she used bleaching creams her whole life to lighten her appearance” because she “did not want to identify with being Indigenous.” Olga’s conversation with her grandmother helped her recognize the cultural *Rifts* that had impacted her family; it also represented a moment of *Reconnection* that is elaborated on later in this narrative.

#### 5.2.2. Longing

Olga’s experiences crossing the border and facing anti-immigrant and anti-Indigenous sentiments have shaped how she relates to the land to which she and her relations belong. Reflecting on the role of land, Olga says:

The land we walk upon [is] our original land. I think it’s the land before borders were placed before countries were named, before territories were seized by force. I think we belong to this land. It’s not about ownership like we own it or this is our land, but rather we are of this land.

Within this space of reflection, she remembers the sacrifices that her parents have made to move her family here and adds that “when I think of my mother and others like her who are migrating, who are coming north again, I feel like they’re just coming back home.” Olga says, “there is a feeling of ancestral desire within us to seek a better place.” Her *Longing* to have a sense of home in the United States parallels her desire to connect with other Indigenous people. She also makes the connection to the continuity of land. She emphasizes that the land she is currently residing on (in an urban area of the American west) is still part of her original land and territory as it was before colonization and the imposition of the nation-state and that movement and migration are natural human processes.

#### 5.2.3. Reconnecting

This *Longing* to belong prompted Olga to seek answers and reconnect to her Indigenous identity and practices. In addition to taking college classes on Mexican history and migration, she became involved in organizations like M.E.Ch.A and Danza Azteca. These experiences allowed her to “encounter different Indigenous people.” Reflecting on the impact of these experiences on her identity, she says:

So then I went to, in college, I was Chicana, right? As an activist. And then wanting to honor that history and the reality of how I grew up. And then eventually again, learning more about who we were as [Yaqui and Otomí] people and claiming that as well.

These acts of *Reconnection* also included reintegrating her previously erased name. For Olga, this was an act of “reclaiming that part of who I am and saying I want to seek my joy.”

After college, Olga continued reconnecting with her roots through traditional healing work and service to the community via non-profit work and activism. She has also reconnected by integrating traditional practices into her personal life, such as hosting an Indigenous naming ceremony for her child:

It’s just putting us on this path of understanding what names mean and the power of that and community… And it was just the most beautiful experience. It was at night. We’d buried the placenta by a tree as, as is part of our tradition so that the tree grows strong and our children can grow strong with it.

#### 5.2.4. Affirmation

Olga affirms her Indigenous identity, saying, “I’m a proud Indigenous woman.” This identity reclamation is a part of her hope for her descendants and the legacy she intends to leave behind. Part of her legacy is envisioned as perpetuating the joy that she found for herself:

So I want to spend…the rest of my time finding joy in the work that I do in community, finding joy through the healing that I’m able to bring to myself and those around me. I owe it to them and to myself and to my future generations to understand what that looks like for me and to just live in a place of joy.

Olga further shares hopes for her community legacy:

I want my legacy to be that we are Indigenous people, that we are unapologetic about that, that we’re very proud that this is our homeland and that we’ve always been here and that will always continue to be here. And I want them to walk with that knowledge, you know, feeling just so connected to their identity and to who they are and that no one can define them but themselves.

In actively considering the generations before and after her, Olga affirms the importance of the identity reclamation that has happened throughout her lifetime. In the process, her activism and community work continuously build toward the future of unapologetic joy and belonging that she dreams of for her community.

### 5.3. Yoloteotl

#### 5.3.1. Rift

Yoloteotl was born in Mexico and identifies as Mexica (Aztec), Chichimeca, and African American from her maternal and paternal sides of the family, respectively. Her maternal grandmother decided to “immigrate from her rural town in Mexico to the city with my great-grandmother” to pursue “better opportunities.” While relocating “out of the land,” Yoloteotl’s grandmother adapted by “becoming a little bit more colonized” and harboring disdain toward Indigenous people from their community. This began a considerable cultural *Rift* that reverberated within Yoloteotl’s family. Yoloteotl’s mother, for example, was “always putting down Indigenous cultures” and felt “ashamed” enough about her Indigeneity that she would dye her hair “into a blondish color.” Yoloteotl grew up traveling between Mexico, the United States, and Canada. Reflecting on her childhood, she recalls “getting a lot of exposure to diversity” and “a lot of cultures” while residing in Canada. However, her family continued to teach her to regard the Indigenous community with contempt.

#### 5.3.2. Longing

Despite the message from her family to push away their indigeneity, Yoloteotl felt a strong sense of disconnection throughout her childhood with what her family told her about Indigenous peoples and their cultural practices. She notes how she “kept looking for something different” and, from the early age of eight, “was trying to be a runaway.” Though her family attempted to “bring me back” to their ways of being, she never identified herself “fully with everything that they were telling me” and longed to “try to always look outside of family.”

As this *Longing* within her grew, Yoloteotl began to “use a lot of other substances” during her teenage years. She says “I wasn’t clean and sober” and “almost died.” Within this period, Yoloteotl had the opportunity to observe and witness “an Aztec dance in Mexico.” While she “never approached them or anything like that,” she “felt a really strong call.” Yoloteotl reflects on how, after having an episode of toxic psychosis, “the drum used to call me a lot.”

#### 5.3.3. Reconnecting

As she watched the Aztec dancers, Yoloteotl felt inspired to re-engage with these Indigenous practices and “start changing my life, start to change my habits.” When she was 19, Yoloteotl witnessed the Aztec dance circle again and approached the dancers; they encouraged Yoloteotl and her grandmother to “come here and dance.” One dancer, who later became “like her grandmother,” adopted her and welcomed her back into their danza traditions. At that point, Yoloteotl expressed gratitude and inquired what the dancers prayed to. One answered that they pray to “The elements… The Earth, the animals, the fire, the water, the things that are sustaining us.” This conversation marked a significant turning point for Yoloteotl, where she took the first step to reconnect with her Indigenous identity and, in return, shared cultural wisdom that continues to guide her way of living close to the land. As she reflects on this moment, Yoloteotl notes, “that’s exactly what I [was] looking for, and this is where I belong. I started my journey from there, and that’s been, like now, 20 years or 21 years. I’m really grateful.”

This first instance of *Reconnection* traces its roots back to Yoloteotl’s childhood when she would unknowingly nurture her connections to her identity by cultivating the land alongside her grandmother, who had a “log with plants” and a “huge and beautiful herbal garden.” Yoloteotl remembers “going out and playing” with the plants in the garden and how “talk[ing] to the plants” and making “little medicines” would “bring a lot of peace and content.” This aspect of her childhood represents an instance of *Reconnection* that arises from ancestral knowledge, which guided her actions long before she was aware of the processes of cultural reclamation.

Later in life, Yoloteotl continued her intentional *Reconnection* with Indigenous practices. For instance, before delivering her child, she traveled to Mexico to receive prenatal care from an Indigenous *partera* (midwife). “[The midwife] used a lot of the herbal medicines with me,” she said. Through working with the midwife, Yoloteotl learned about the practice of the *cuarentena*, an Indigenous tradition of Mexico in which post-partum parents intentionally spend 40 days focusing on slow and still time to connect with their babies (Waugh 2011). Yoloteotl further describes:

We didn’t accept anybody outside of the house for 40 days, only really close friends and family.… It’s just you and your baby, and somebody must take care of you for 40 days. The midwife with my son, she was like you don’t even have to get up. You cannot even get up. [My husband] had to carry me on his back to the Temazcal, to the sweat lodge, and I had to bathe there. You are not allowed to go outside, get any cold air or anything like that. Just to let all your organs and your bones for the baby to be able to be grounded into this dimension.

As Yoloteotl deepened her connection to Indigenous practices, she was not only able to ground herself within the culture but also provide solid ground for her child.

#### 5.3.4. Affirmation

Yoloteotl’s path from *Reconnection* to *Affirmation* is deep-rooted with her connection to land, plants, and family. “My family is the plants, the animals, and everything that is here on this land,” she says. “Everything is, it’s in relation to me, and I’m in relation to what is given.” Yoloteotl affirms her identity by employing critical approaches to her community-based work of serving the women and working on the land in her community. Yoloteotl illustrates her vision of *Affirmation* as:

Being able to continue to heal, to learn, to adapt the education, and the notes that I’m acquiring through the educational system… to create change into the systems of oppression, to help our Indigenous communities to thrive.

Yoloteotl affirms her identity by remembering the “importance of our contact and the reconnection” with the ancestors and the elements. Within this memory work, Yoloteotl envisions her ancestors as her grandparents, the mountains, the plants, and the elements because “we’re coming from them.” This commitment evokes her role to help others “connect back to the land, be more aware, bring that awareness with women, with men, and help women heal the wounds with the men.” Sharing her vision for her community, she describes how Indigenous ways of knowing and being are finally (though perhaps slowly) recognized as legitimate knowledge and practice systems. She says, “Everything that we carry as Indigenous people is finally starting to be recognized,” not as negative stereotypes of people talking to plants and nature, “But instead, as a decolonizing [approach] to open heart thinking. One of my roles… with time, is to bring awareness to our people. The importance to honor our ancestors.”

Yoloteotl’s dedication to her community extends back to her childhood—when she was a child, she always said, “I’m going to change the world. It’s what I want to do.” Her commitment emerged after years of learning and growing, partly through her access to educational systems such as community college and graduate school and from having the courage to reconnect to her Indigenous roots and the faith required to maintain it for her community and future generations.

## 6. Discussion

In this paper, we propose a conceptual model of identity formation based on cultural reclamation among three Indigenous Mexican individuals living in the United States. This model identifies a dynamic process that our co-researchers connect to their personal healing and community resilience, as shown in the three case narratives. Our model contributes to Indigenous Latinx cultural identity literature by presenting a process of identity reclamation that prioritizes healing, illustrates both individual experiences, and acknowledges the importance of community *Affirmation*. Rather than focusing solely on the individual, we demonstrate how individual experiences are deeply embedded in the collective, and individual healing cannot be separated from the healing of the whole.

This model aligns with literature that describes the critical role of enculturation, cultural reclamation, and positive cultural identity in Indigenous health and well-being (Beltrán et al. 2020; Beltrán et al. 2023; Evans-Campbell 2008; Fernandez 2019; Fernandez and Beltrán 2022; Gone 2011; Masotti et al. 2023; Walters and Simoni 2002). The reflection (ocean current-driven) component illustrates how remembering can activate generative meaning-making and integration of past experiences in an individual’s lifetime and in previous generations. By emphasizing the role of memory work in the process of identity reformation, this model confirms the importance and healing impact of memory work in identity healing and reformation for diasporic Indigenous people (Clarke and Yellow Bird 2021; Hernández-Ávila 1995; Moraga 2011; Zepeda 2022).

Because reflection animates the model, the pathway through the model is non-linear and organic, much like water itself. Although we present our model in a linear arc through these narratives, co-researchers often move through the model in a non-linear order, sometimes skipping stages, sometimes moving through cycles multiple times, and sometimes even being in multiple zones at once. For instance, Bonifacio’s period of being drawn to the “cholo lifestyle” as a teenager could indicate both *Longing* for an identity that he could belong to, as well as a culmination of the land, cultural, and social *Rifts* that occurred in his family. Similarly, Yoloteotl and Olga’s decision to engage in traditional birthing and naming practices can count as both a *Reconnection* to Indigenous cultural practices and *Affirmation* due to their impact on future generations.

Water shapes and molds, and our model also shapes dynamic ways of thinking about identity reclamation. With its fluid nature, our model centers healing while illustrating a non-linear pathway that one must attend to across an entire life course. Thus, it centers on Indigenous ontologies of cyclical time (Aikenhead and Ogawa 2007; Kidman et al. 2021) and diverges from Western colonial notions of healing as a linear path with an endpoint. The model further illustrates how identity cannot unfold solely within an individual due to the importance of community and relationality—the end goal is to be together, to unite, and to heal as individuals within the collective community, as shown in the dually positive impact connecting with community has had on each co-researcher.

Further insights from this model can be gleaned from looking more closely at the similarities and differences among each case narrative. The similarities begin with how Bonifacio, Olga, and Yoloteotl all named significant historical and recent *Rifts*. Internalized suppression and shame about Indigenous identity (Caminero-Santangelo 2004; Sotero 2006) are reflected in how Yoloteotl and Olga’s mothers dyed their hair and bleached their skin, respectively, to hide their indigeneity. *Linguistic Rifts* occurred for Bonifacio, who needed to learn new languages constantly as his family moved to different parts of Mexico and the United States, and for Olga, who needed to remove part of her name. Their experiences highlight how educational systems can reinforce the erasure of Indigenous identity (Barillas Chón et al. 2021). All three narratives highlight how larger capitalist and colonial forces led to land *Rifts* that separated self from culture.

The narratives also show how formal education, particularly college, played a major role in *Reconnection*. Olga’s college class on Mexican history catalyzed the opportunity for her to interview her grandmother, which simultaneously served as an instance of discovering the *Rifts* in her family and reconnecting to her Indigenous identity. For Bonifacio and Olga, M.E.Ch.A also served an important role in introducing them to an activated and mobilized Indigenous community for the first time. Even though neither of them identifies as Chicana/o/x, involvement with this student group opened a door for them to explore and more deeply connect to their own Indigenous communities. Their experiences note higher education’s impact in helping Indigenous people reconnect to and affirm Indigenous identity (Kovats Sánchez 2020) and this additional role of student clubs and organizations within higher education settings.

### Limitations and Future Directions

There are limitations to our conceptualization. First, this manuscript highlights a small subsample of the larger study community. Second, we acknowledge that this exploration arrives at a time when there is contestation about definitions of Indigenous identity in the United States and Mexico alongside ongoing violent settler colonial projects for tribes in both countries that have very real impacts on the people still residing in their original territories. In this article, we are not making the case for establishing a process that could lead to a pan-Indigenous identity. Rather, we highlight these three stories as they illustrate a process of healing, connection, and integration of self into the community to disrupt the impacts of settler colonial violence on displaced diasporic Indigenous peoples. Indeed, we are responding to the call for deep localization by describing and amplifying the specific stories of these people within this specific community of urban diasporic Indigenous peoples of Mexico as they articulate and experience their own Indigenous identities.

To address these limitations, additional research can expand this conceptual model to a larger sample, possibly among other Indigenous subgroups not limited to Indigenous peoples of Mexico. After having presented this model, we intend to systematically analyze it within the rest of our data for future research. Further endeavors that create spaces of learning, exchange, and dialogue for Indigenous peoples of Mexico living in the United States would support the emergent and growing knowledge base and should occur beyond traditional academic spaces and forms. Finally, there is limited space to build additional nuances of ways in which this identity model will ebb and flow at different times of our lives, encouraging further exploration of this model via additional methodologies such as autoethnography to illustrate how this model illustrates lived experience.

## 7. Conclusions

This paper presents a dynamic conceptual model of identity (re)formation that honors the survivance of Indigenous Mexican people living in the United States. The model illustrates how Indigenous Mexican identities can be repaired, reclaimed, (re)formed, and sustained by moving through the model stages of *Rift* (disconnection from land, culture, and community), *Longing* (yearning to find what was lost), *Reconnecting* (reclaiming cultural practices), and *Affirmation* (strengthening of identity through community) via *Reflection* (memory work). The case narratives that highlight the model showcase how organic, cyclical, and healing this process of identity reformation can be. This conceptual model may be helpful in further understanding identity development and healing among Indigenous Latinx communities.

We close with gratitude for all that water has taught us. Like water, we are responsive. We can heal, nourish, and replenish ourselves. Though we are shaped and molded in different forms, we remain the same. Even if you cannot see us, we are there, resurging. Like water, we re-member.

## Data Availability

Data presented here are available upon request from the second author.

## Footnotes

1 We use this spelling of “re-membering” to describe the process of both remembering cultural knowledge and re-membering in the sense of putting back together in a way that reflects the integration of traditional Indigenous knowledge with one’s identity.

2 We are using the term “co-researchers” to indicate that participating community members were not only part of sharing their stories as data but were also actively involved in the design, development, implementation, and ongoing oversight of our project. As scholars committed to Indigenist research, we actively seek to disrupt the hierarchical and extractive nature of research. We believe this term better identifies their roles as collaborators and conspirators in the liberatory aims of our project.

3 While not all diasporic Indigenous peoples from Latin America residing in the U.S. identify as Latinx, we are using this term because Latinx is the broadest category/pan-ethnic category most commonly used to refer to people from Latin America.

4 While our model reflects many qualities of the ocean, in particular the layers of the ocean, we are making this connection through metaphor and not with literal parallels to the ocean layers in their myriad complexity.

5 The cholo lifestyle to which Bonifacio refers marks a distinct subculture that has been described as having characteristics such as “defiant individualism,” *carnalismo* (brotherhood), and machismo which some youth may adopt as a way to form a community when dominant society continuously marginalizes them (Valdez 2003).
